# Determination of the NO_x_ Loading of an Automotive Lean NO_x_ Trap by Directly Monitoring the Electrical Properties of the Catalyst Material Itself

**DOI:** 10.3390/s110908261

**Published:** 2011-08-25

**Authors:** Peter Fremerey, Sebastian Reiß, Andrea Geupel, Gerhard Fischerauer, Ralf Moos

**Affiliations:** Bayreuth Engine Research Center (BERC), Universität Bayreuth, 95440 Bayreuth, Germany

**Keywords:** on-board diagnosis, emission control, TWC, DPF, cavity perturbation

## Abstract

Recently, it has been shown that the degree of loading of several types of automotive exhaust aftertreatment devices can be directly monitored *in situ* and in a contactless way by a microwave-based method. The goal of this study was to clarify whether this method can also be applied to NO_x_ storage and reduction catalysts (lean NO_x_ traps) in order to obtain further knowledge about the reactions occurring in the catalyst and to compare the results with those obtained by wirebound NO_x_ loading sensors. It is shown that both methods are able to detect the different catalyst loading states. However, the sensitivity of the microwave-based method turned out to be small compared to that previously observed for other exhaust aftertreatment devices. This may limit the practical applicability of the microwave-based NO_x_ loading detection in lean NO_x_ traps.

## 1. Introduction

Increasing fuel costs and the need to reduce CO_2_ emissions are the main drivers for the market penetration of fuel-efficient leanly operated internal combustion engines in the field of passenger cars. Due to their lean operation mode, NO_x_ removal with a conventional three-way catalyst is not possible [1]. At the same time, the emission limits for NO_x_ have been strongly tightened. Besides the ammonia-selective catalytic reduction process, in which NO_x_ is selectively reduced to nitrogen and water even under lean conditions [2], the NO_x_ storage catalyst (abbreviated NSR, also often denoted as lean NO_x_ trap, abbreviated as LNT) has been developed [3], especially for direct injection gasoline engines that operate in the lean mode. During a lean phase, NO_x_ is oxidized, absorbed, and stored in the form of nitrates. Once NO_x_ cannot be stored anymore, a short rich period follows, in which the formed nitrates decompose and the reduction of the released NO_x_ occurs. By now, the LNT concept has been applied also for diesel exhaust gas aftertreatment [4], and the SCR system has been discussed for leanly operated gasoline engines [5]. Even the combination of both NO_x_ abatement techniques, with ammonia being formed during the short rich phase in the LNT, is an in-series application [6].

The NO_x_ storage catalyst devices consist of ceramic cordierite honeycomb structures coated with fine-grained oxides to increase the open surface [7,8] and with catalytically active materials, namely platinum group metals, with alkaline (-earth) oxides or carbonates, and with oxygen storage components, typically ceria-zirconia solid solutions [9,10]. On the surface of the platinum group metals, NO molecules are oxidized to NO_2_, which also get oxidized and stored on the alkaline (-earth) based storage sites in the form of nitrates. Equations (1) and (2) reflect this for a barium-based LNT formulation:

(1) $$\text{NO }+\;½\;O_{2}\to {\text{NO}}_{2}$$

(2) $${\text{BaCO}}_{3}+2\;{\text{NO}}_{2}+½O_{2}↔\text{Ba}{\left({\text{NO}}_{3}\right)}_{2}+{\text{CO}}_{2}$$

Just before the storage capacity of a NO_x_ storage catalyst is exhausted and the catalyst begins to let NO_x_ pass, the engine is switched to the rich operation mode and the stored NO_x_ species are reduced by the platinum group metals to N_2_ [10].

In order to take full advantage of the provided NO_x_ storage capacity, LNT based exhaust gas aftertreatment catalysts usually cannot be operated in an open loop. Therefore, a NO_x_ sensor is mounted downstream of the LNT to detect NO_x_ breakthroughs and to act as a part of a closed-loop control system, helping to not overload the LNT. In addition, a λ-probe ensures that the regeneration period is only as long as necessary, since otherwise CO or hydrocarbon breakthroughs may occur [11].

This makes clear that appropriate exhaust gas sensors become more and more important, not only for engine control, but also for OBD (on-board diagnosis) purposes [12,13]. Besides the direct measurement of the exhaust gas component NO_x_ or of the normalized air-to-fuel ratio λ, it would be beneficial for an optimized control to know the degree of catalyst loading [14]. In the past few years, R&D focused in two directions. On the one hand, two conventional types of NO_x_ gas sensors have reached a maturity for serial production [15,16], on the other hand, in research labs initial work is being conducted to measure directly the status of an exhaust gas catalyst—without the detour to the exhaust gas concentration [17].

This direct status detection method can be divided in a wirebound and a contactless microwave-based approach [14]. With respect to the wirebound technique, it has been shown that, by measuring the electrical impedance of the coating material itself *in situ* in a frequency range from 20 Hz to 1 MHz, the state of an LNT with respect to NO_x_ loading, regeneration, sulfurization (poisoning), and thermal aging can be determined [18,19].

The microwave-based approach has been suggested recently to determine quantitatively the degree of oxygen loading in three-way catalysts [20] and to quantitatively measure the soot mass of diesel particulate filters [21]. When storing gas in the catalyst or when soot is deposited on the filter walls, the electrical properties such as the conductivity of the catalyst material change. Electromagnetic waves propagating inside the metallic exhaust pipe are influenced by this conductivity change. This leads, for instance, to the perturbation of a cavity resonator, which is the fundamental effect of the microwave-based catalyst monitoring method [22]. With oxygen or soot loading, the electrical conductivity changes by decades leading to marked effects in the microwave absorption. Very recently, this method has been successfully proven to determine the ammonia loading of zeolite-based SCR catalysts [23], in spite of far lower conductivity changes in this case [24]. Another very interesting related approach is the use of the microwave technique in combination with sensitive layers for gas sensing purposes [25].

For LNT systems, one has to expect only small conductivity changes with NO_x_ loading [19]. In addition, one has to distinguish between NO_x_ loading and oxygen loading, the latter occurring in the ceria-zirconia components of the commercial LNT coating formulation. However, preliminary results (Figure 7 in Reference [17]) indicate that it might be possible to use the microwave-based approach also for LNT control.

In this study, we compare the wirebound and the microwave-based catalyst state observation techniques directly. The paper is structured as follows: first, the microwave-based approach is introduced and the results are discussed. Second, the results obtained with several wirebound sensors installed in line in the LNT device along the gas flow axis are shown and discussed. In a third section, the results obtained simultaneously from both methods under special NO_x_ loading cycles are shown and compared in the light of the NO_x_ storage process in the catalyst. One difference in the methods is obvious from the outset: while a wirebound NO_x_ loading sensor measures the NO_x_ loading locally (spatially resolved), the microwave-based technique integrates over the entire catalyst. It also an aim of this study to evaluate whether the microwave-based technique has the potential for a serial application in lean NO_x_ traps for NO_x_ abatement in the automotive exhaust.

## 2. Microwave-Based Approach

The microwave-based approach exploits the perturbation of a microwave cavity resonator. The approach is similar to the ones that are widely used for determining the dielectric material properties at microwave frequencies [26]. In contrast to the conventionally applied method, it is not the perturbation of a previously empty cavity by a small material sample that is considered here, but rather the perturbation of the entire LNT material-filled cavity by changes in the electrical material properties of the catalyst itself.

### 2.1. Microwave-Based Approach: Experimental

**Figure 1.**
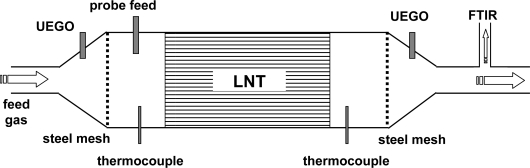
Schematic test setup for the microwave-based measurements.

All measurements were conducted in a lab test bench for catalysts. Figure 1 shows the setup schematically. The barium-based LNT device was mounted in a stainless steel housing. The dimensions of the LNT device were 118 mm × 124 mm (diameter × length). The housing was flange-connected with two cones to the gas feed (synthetic exhaust gas) and to the vent. Upstream and downstream of the LNT, wideband λ-probes (UEGO sensor, for details see [27]), and thermocouples (type K) were mounted in the catalyst housing as shown in Figure 1. The UEGO sensors measure the normalized air-to-fuel ratio λ in a wide range.

The steel canning of the LNT acts as a cylindrical electromagnetic waveguide. A short stub antenna serving as a microwave probe feed (as described in [28]) was also installed. The resulting microwave one-port element was connected with an automatic vector network analyzer by coaxial lines. To obtain a precisely defined cylindrical cavity resonator, gas permeable steel meshes (mesh width: 4 mm) were inserted on both sides of the catalyst (for details see [28]). The distance between the meshes was 375 mm. The entire test bench was electrically heated to an operation temperature between 300 °C and 350 °C at a gas flow of 20 L/min. The test bench had two different gas lines to switch very rapidly from lean to rich feed gas. A part of the outlet gas was pumped into a Fourier transform infrared (FTIR) spectroscopy analyzer measuring the concentrations of the optically active gas components downstream of the LNT. The canned LNT is characterized by its frequency-dependent input reflection factor:

(3) $${\underline{S}}_{11}(f)={\underline{b}}_{1}(f)/{\underline{a}}_{1}$$

where *a*_1_ and *b*_1_ denote the complex amplitudes of the electromagnetic waves incident on and reflected off the one-port cavity resonator. Further details of this method are given in [22] and [28].

**Figure 2.**
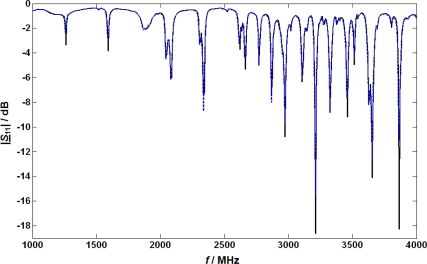
Negative return loss, or logarithmic absolute magnitude of the reflection coefficient |*S*_11_|, as function of the frequency in the unloaded lean state (solid curve) and the NO_x_ loaded state (dashed), both at 300 °C.

For the basic tests of the microwave system, the LNT catalyst was heated to 300 °C, and the LNT was regenerated using 1% H_2_, 100 ppm C_3_H_8_, 5,000 ppm CO and 8% H_2_O in N_2_. Then, a lean base gas without NO or NO_2_ was applied to the LNT (5% CO_2_, 1% O_2_, and 8% H_2_O in N_2_). After the UEGO sensor downstream of the LNT indicated a fully oxygen loaded state of the catalyst, a microwave spectrum was taken in the frequency range from 1,000 to 4,000 MHz to record the properties in the lean but completely NO_x_ depleted catalyst (due to the absence of NO). The absolute value of the obtained reflection parameter |*S*_11_| is plotted (in dB; solid curve) versus frequency *f* in Figure 2. Depending on the applied frequency, resonance effects occur recognizable by the dips at distinct frequencies. Then, 4,000 ppm NO were added to the base gas and when the NO_x_ concentration downstream of the catalyst reached the NO inlet concentration, a second basic spectrum of *S*_11_ was taken. The absolute value of |*S*_11_| in this state is also plotted in Figure 2 (dashed). At first glance, the results seem disappointing since both curves look very similar. However, a closer look shows that a small shift in the resonance frequencies and also a very slight broadening of the minimum peak occurs, but both effects are far less pronounced than in the previously investigated three-way catalysts [20] or diesel particulate filters [21]. The fractional changes in the resonance frequency due to NO_x_ loading Δ*f*_res_/*f*_res_ were compared for all resonance frequencies and the resonance frequency with the highest effect of NO_x_ was used for further studies. The following first study was conducted at around 2,615 MHz. Due to system modifications (e.g., the addition of the NO_x_ sensors and a different catalyst temperature) the optimum frequency for further tests with NO_x_ sensors included was found to be about 3,500 MHz.

### 2.2. Microwave-Based Approach: Results and Discussion

The time-dependent tests were carried out as follows. At the beginning of the test run, a lean base gas without NO_x_ was applied to the LNT (5% CO_2_, 1% O_2_, and 8% H_2_O in N_2_) at *t*_1_ ≈ 0.5 h after a previous rich regeneration phase (1% H_2_, 100 ppm C_3_H_8_, 5,000 ppm CO and 8% H_2_O in N_2_) to fully remove stored NO_x_. Starting at *t*_2_ ≈ 2.5 h, 2,000 ppm NO were added to the base gas for 2 h. The concentration of NO in the feed gas was increased to 4,000 ppm at *t*_3_ ≈ 4.5 h. At about *t*_4_ ≈ 6.5 h the NO concentration was switched back to 2,000 ppm, and after *t*_5_ ≈ 8.5 h, the feed gas consisted of only base gas without NO. During the whole measurement, the catalyst temperature was kept constant at 300 °C.

**Figure 3.**
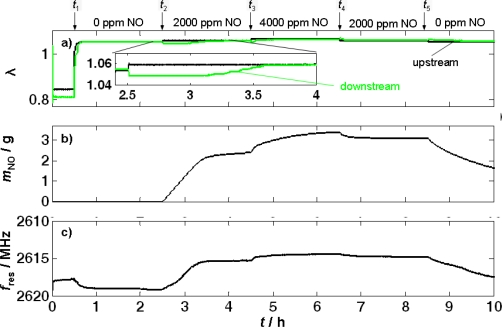
NO_x_ loading experiment of an LNT monitored by the microwave-based technique at 300 °C. Time responses of **(a)** the normalized air-to-fuel ratio λ upstream and downstream of the catalyst, **(b)** the NO_x_ loading as calculated by Equation (4) and **(c)** the resonance frequency *f*_res_.

First, the λ values obtained by the UEGO sensors upstream and downstream of the LNT during this test run are compared in Figure 3(a). At *t* = *t*_1_ the feed gas switches from the rich to the lean composition. The UEGO sensor upstream of the catalyst reflects this fast switch, whereas the UEGO sensor downstream has a slightly delayed response. Its signal remains around λ ≈ 1 for several minutes before it reaches the same value as the sensor upstream of the catalyst. The delay is caused by the oxygen storage component of the LNT formulation. As soon as the LNT is completely oxygen loaded, both UEGO sensor signals coincide. Starting from *t* ≈ *t*_2_, NO is added to the feed gas. The trace of the UEGO sensor signal, as highlighted in the inset, mirrors the NO_x_ storage behavior. As soon as NO is added to the gas mixture, λ of the feed gas increases a little. In contrast, λ downstream of the LNT drops a bit at the same time. This is not surprising, since according to Equations (1) and (2), oxygen is required for the storage reaction and therefore, as long as the LNT stores NO_x_, λ downstream of the LNT should be lower than without NO in the feed. At about 3.5 h, the catalyst is saturated (for 2,000 ppm NO in the feed), and no NO_x_ can be stored anymore, *i.e.*, there is no more oxygen consumption, and λ downstream of the LNT reaches the feed gas value again. A similar behavior occurs after *t*_3_, when the NO concentration is further increased. Due to the chemical equilibrium of the storage reaction, the storage capacity of the LNT is greater at higher NO gas concentrations and more NO_x_ can be stored. Analogously, the equilibrium changes back to lower loading states when NO is decreased to 2,000 ppm again at *t*_4_ (6.5 h) as well as to 0 ppm at *t*_5_ (8.5 h). In both cases NO_x_ desorbs from the LNT.

These storage and release processes are proven by the gas analysis downstream of the LNT (not shown here). NO_x_ was not detected by the FTIR spectrometer until about 3.1 h, *i.e.*, the LNT stored all offered NO beginning at *t*_2_ (2.5 h) until NO_x_ slip occurs at about 3.1 h. The storage capacity is exhausted at about 3.5 h, when NO_x_ downstream of the catalyst reaches a constant level and the λ values upstream and downstream of the LNT begin to coincide. With increasing NO concentration in the feed gas, additional NO_x_ storage occurs, indicated by a difference in the λ values and a slightly delayed increase of the FTIR spectroscopy signal. Also, the delayed decrease of the NO_x_ concentration in the outlet gas (measured by the FTIR spectrometer) after changing back to 2,000 ppm NO and 0 ppm NO reveals a loss of NO_x_ in the LNT.

Balancing the nitrogen oxide concentrations upstream and downstream of the LNT during the loading period should yield information on the stored amount of NO_x_ in the LNT. The total stored NO mass, *m*_NO_, can be calculated from the volumetric flow rate *V* and the NO density *ρ*_NO_ by Equation (4). The NO_x_ concentration in the outlet obtained from the FTIR spectroscopy, *c*_NOx,FTIR_, is subtracted from the NO concentration added to the gas flow, *c*_NO_. The integration yields the stored mass of NO:

(4) $$m_{\text{NO}}=\rho_{NO}\int \dot{V}\left(c_{\text{NO}}-c_{\text{NOx},\text{FTIR}}\right)\mathrm{dt}$$

The calculated stored amount of nitrogen oxide during this test is shown in Figure 3(b). It meets the expectations and indicates a constantly increasing *m*_NO_ due to complete storage of the dosed NO between *t*_2_ = 2.5 h and the beginning of the NO_x_ slip in the outlet at 3.1 h. The increased NO concentration in the feed gas for *t*_3_ < *t* < *t*_4_ goes along with a slightly increased stored amount of NO. The corresponding opposite behavior can be observed, when the NO feed gas concentration is lowered to 2,000 ppm at *t*_4_ = 6.5 h and the LNT releases NO_x_. After *t*_5_ = 8.5 h (0 ppm NO in the feed), the loading level decreases further due to NO_x_ desorption from the LNT.

The same picture is given by the microwave resonance frequency, *f*_res_, in Figure 3(c) (note that the ordinate axis points downward to facilitate the comparison between the resonance frequency curve and the curve for the total stored NO mass, *m*_NO_). One can see clearly the difference of the signal between the rich and lean gas atmospheres soon after *t*_1_ at the beginning of the experiment. This is caused by the oxygen storage capability of the LNT formulation. As known from three-way catalysts, the oxidation state of ceria (as an oxygen storage component) has a major influence on the microwave absorption [28]. With the beginning of the NO_x_ storage at *t*_2_, *f*_res_ decreases constantly until about 3.3 h. From then on, it remains constant. The curve strongly resembles the NO mass loading curve in Figure 3(b). A further increased NO concentration in the feed gas (*t*_3_ < *t* < *t*_4_) also affects the microwave signal, indicating an increasing mass of stored NO in the LNT. Switching back to 2,000 ppm NO at *t* = *t*_4_, the resonance frequency shifts a little, reflecting the NO_x_ release. Both, the curves of the calculated mass of stored NO and of the resonance frequency show that the second phase with 2,000 ppm NO results in a higher NO_x_ loading compared to the first phase with the same concentration between *t*_2_ and *t*_3_ (3.5 h and 4.5 h). The desorption of NO_x_ after switching to base feed gas without NO at *t*_5_ = 8.5 h is indicated by an increasing resonance frequency and therefore a lowered NO_x_ loading level.

In summary, the results of this experiment give a first hint that the data obtained from the microwave-based approach indicate the NO_x_ loading state of the LNT catalyst. The results coincide with the gas analysis in the outlet. This indicates that the NO_x_ loading of the LNT can be measured directly by the contactless microwave-based monitoring technique.

## 3. Wirebound Impedimetric Sensor Approach

This section deals with the setup of the impedimetric sensors as well as with basic impedimetric measurements of one sensor sample. This is a precondition for the subsequent section, in which we report on the *in situ* observation of the entire LNT device by several impedimetric sensors along the catalyst combined with the microwave-based technique.

### 3.1 Wirebound Impedimetric Sensor Approach: Experimental Setup

**Figure 4.**
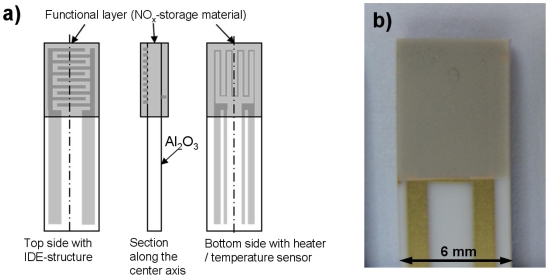
Setup of a single impedimetric sensor. **(a)** Sketches of different perspectives of the sensor element consisting of an alumina substrate, interdigital electrodes and a heater structure coated with the sensitive layer. Please note that the functional film on both sides is identical with the LNT material of the used catalyst. **(b)** Photograph of the top side of a sensor.

In order to directly measure the electrical properties (especially the electrical conductivity σ) of the NO_x_ storage material *in situ* while NO_x_ is sorbed or released, impedimetric sensors with the catalyst material as sensitive layers were constructed. Figure 4(a) shows sketches of the setup of the NO_x_ loading sensor; Figure 4(b) is a photograph of one of the sensors employed. Alumina substrates (purity 99.6%) with the dimension of 51 mm × 6 mm and a thickness of 630 μm served as sensor substrates. At first, a platinum heater structure was screen-printed on the bottom side of the sensor and was fired at 1,200 °C for 20 min. The gold interdigitated electrode (IDE) structure on the top side was also screen-printed. Both the distances between the gold interdigitated electrodes and their width were 50 μm. The gold structures were fired at 850 °C for 20 min. Afterwards, an electrically insulating protection layer was screen-printed on top of the heater structure and fired at 850 °C for 2 h. Finally, the transducer was coated with the lean NO_x_ trap (LNT) material itself. This material is identical with the LNT catalyst formulation, hence the sensitive layer represents the catalyst material itself. To deposit the LNT functional layer on the IDE structure, the sensors were dipped into the slurry consisting of the LNT powder mixed with distilled water. The LNT coated substrates were fired at 600 °C for 6 h. The sensors were degreened in lean-rich cycles for several hours to obtain stable signals for reproducible measurement results.

### 3.2 Wirebound Impedimetric Sensor Approach: Basic Results

In [19] and [29] it was found that the electrical conductivity of NO_x_ storage materials at the operation temperature of several hundred °C depends on the state of the catalyst. These changes in the complex impedance *Z* can be analyzed via impedance spectroscopy.

**Figure 5.**
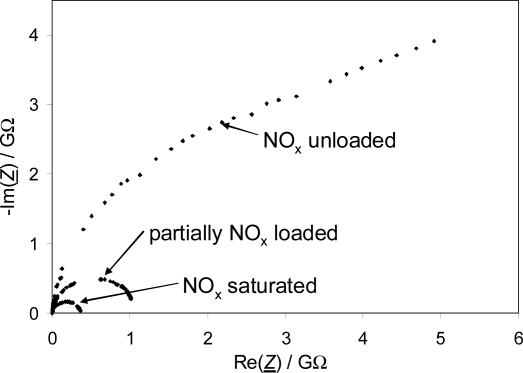
Impedance of an impedimetric sensor (see Figure 4) when loaded with different amounts of NO_x_ at 350 °C and gas compositions at a gas flow of 2 L/min: 10% O_2_, 5% CO_2_, 2% H_2_O for loading with 12 ppm NO and 12 ppm NO_2_ in N_2_

The measured impedance spectrum of a sensor coated with the LNT formulation reveals a semi-circle in the complex plane (-Im(*Z*) vs Re(*Z*)), at least if a little NO_x_ is stored in the material. This is the typical appearance of an equivalent parallel circuit consisting of a resistor and a capacitor (Figure 5). The dielectric permittivity of the alumina substrate and to some extent of the LNT material contribute to the capacitance *C*. If the conductivity of alumina could be neglected, the measured resistance *R* would reflect the conductivity of the LNT material, which depends on its NO_x_ loading state.

At first, before installing the impedimetric sensor device in the catalyst housing, the functionality of each single sensor was tested in a lean gas mixture (10% O_2_, 5% CO_2_ and 2% H_2_O in N_2_) at 350 °C. The complex impedance of the LNT material was observed while loading the storage sites with 24 ppm NO_x_ (12 ppm NO and 12 ppm NO_2_). As an example, an impedance plot in the complex plane for different NO_x_ loading states of one sensor is shown in Figure 5. Obviously, the sensor behaves as expected. Especially with increasing NO_x_ loading, semicircles appear. With progressing NO_x_ loading, the diameters of the semicircles, reflecting *R*, decrease clearly, which means that the conductivity of the material increases with NO_x_ loading. In the unloaded state, however, the very high resistance of the LNT material may not be measured correctly. The resistance *R* in the NO_x_ unloaded state gives a value in the range of 10 GΩ. Taking into account the geometry of the electrodes, a rough estimation of the resistivity using the geometry factor defined in [30] gives a value of about 10^11^ Ωcm. For comparison, at 350 °C, the resistivity of 99.6% alumina was determined to be about 10^14^ Ωcm by reference [31]. Therefore, the conductivity of the alumina substrate should have only a minor influence in the unloaded state. With increased NO_x_ loading, the conductivity of the LNT-material increases also and the influence of the alumina substrate can be fully neglected.

## 4. Combination of Wirebound and Microwave-Based Method

With the microwave-based approach it was found that the loading state of the whole catalyst volume can be monitored, since the signal correlates with the average electrical properties whereas one can observe the change in the conductivity with a certain spatial resolution by impedimetric sensors with a catalyst coating. In this section, both techniques will be combined to obtain further information about the catalyst system.

### 4.1. Combination of Wirebound and Microwave-Based Method: Experimental Setup

**Figure 6.**
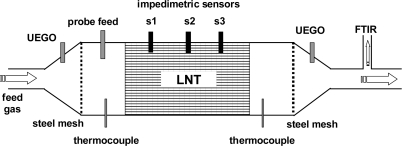
Setup for the combined wirebound and microwave-based approach to determine the average and spatially resolved NO_x_ loading of an LNT.

For the combined wirebound and microwave-based experiments, the test setup shown in Figure 1 was modified so that three impedimetric NO_x_ loading sensors could be installed equidistantly in the housing of the catalyst. This expanded setup is shown in Figure 6. Each sensor was coated with a catalyst-identical film, so the impedance of each sensor reflects the NO_x_ loading state at the corresponding position. The distance between each of the three sensors was 31 mm. Test cycles consisting of lean NO_x_ loading and rich regeneration cycles were applied to the LNT catalyst. The results obtained with the FTIR spectroscopy are compared with the overall signal of the microwave-based technique and the locally resolved responses from the NO_x_ loading sensors mounted along the gas flow axis of the LNT.

The entire test bench was heated to an operation temperature of 320 °C at a gas flow rate of 20 L/min. Either a lean base gas mixture of 5% CO_2_, 10% O_2_, and 2% H_2_O in N_2_ or a rich gas mixture (for regeneration) of 5% CO_2_, 1.5% H_2_ and 2% H_2_O in N_2_ was applied. The sensor in the middle of the catalyst volume (sensor S2) was measured with an impedance analyzer at a frequency of 1 kHz and an amplitude of 1 V. The resistance, *R*, of the sensitive layer was calculated from the impedance assuming an *R*||*C* equivalent circuit. For the sensors S1 and S3, the resistance was determined by measuring the voltage drop over the sensor by a voltage divider with a serial resistance of 1 MΩ and an applied dc voltage of 10 V. Thus, a digital multichannel voltmeter could be used instead of several impedance analyzers. Additionally, other sensor signals like the UEGO sensor and the thermocouples could be measured at the same time with the multichannel voltmeter.

The measured resistance and the NO_x_ sensitivity of the sensors depend on the exact geometry of the sensitive layer and therefore vary little from sensor to sensor. For a better comparison of all sensor signals, a normalized sensor response *A* is introduced by:

(5) $$A=\frac{\text{lg}\frac{R_{\mathrm{unloaded}}}{R}}{\text{lg}\frac{R_{\mathrm{unloaded}}}{R_{\mathrm{saturated}}}}$$

Here, *R*_unloaded_ and *R*_saturated_ denote the resistances in the unloaded and the NO_x_ saturated states, respectively. By relating the resistance change due to NO_x_ loading to the maximal resistance change in the saturated state, the normalized sensor response *A* is always in the range of 0 and 100%.

### 4.2. Combination of Wirebound and Microwave-Based Method: Results

**Figure 7.**
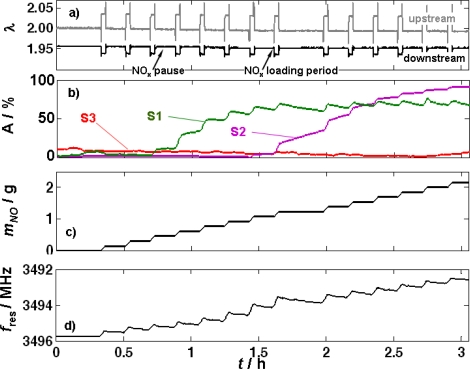
Time dependent results of a stepwise NO_x_ loading experiment. Experimental data: 320 °C; 14 pulses with 2,750 ppm NO_x_ for 120 s each (NO_x_ loading), followed by NO_x_ pauses of 540 s. **(a)** λ upstream and downstream of the catalyst, measured by the UEGO sensor. **(b)** Signals of the impedimetric catalyst sensors (S1, S2, and S3) mounted equidistantly in the catalyst volume. **(c)** Calculated NO_x_ loading according to Equation (4). **(d)** Microwave cavity resonance frequency.

In the first experiment, the fully regenerated catalyst was loaded stepwise with a mixture of NO_2_ and NO. In Figure 7, the recorded data of this measurement are shown: the λ signals upstream and downstream of the catalyst [Figure 7(a)], the NO_x_ loading sensor signals [Figure 7(b)], the amount of stored NO [Figure 7(c)], calculated from of the outlet NO_x_ concentration determined by FTIR spectroscopy), and the curve of the resonance frequency [Figure 7(d)]. 2,750 ppm total NO_x_ (consisting of 2,000 ppm NO and 750 ppm NO_2_) were added to the base gas for 120 s (labeled as NO_x_ loading period) followed by a NO_x_-free interval for 540 s (denominated as NO_x_ pause). This procedure was repeated several times to load successively the catalyst with NO_x_.

The sensor signal of the UEGO sensor upstream of the catalyst, displayed in Figure 7(a), acts as an auxiliary indicator for the presence of a NO_x_ loading phase or a NO_x_ pause. After the eighth loading phase (at about 1.7 h) a longer lasting NO_x_ pause is eye-catching. It was intended to study the effects of a longer period without NO_x_ on the loading sensor signals. As already described in Section 2.2, λ downstream of the catalyst is lower in the presence of NO_x_ due to the consumption of O_2_ in the NO_x_ storage process. The catalyst was able to store all the offered NO_x_ in this experiment as no NO_x_ slip occurred, which was proven by the NO_x_ concentration data in the outlet taken by the FTIR spectrometer (not displayed here). λ downstream of the catalyst [Figure 7(a)] reflects this as well.

The normalized sensor response of the first loading sensor (S1) was the first to increase after introducing NO_x_ to the feed gas [Figure 7(b)]. Already during the third NO_x_ loading phase, its signal started to increase with each single NO_x_ loading pulse. This is in accordance with the results from the pretests - the conductivity of the catalyst material is increasing in the presence of NO_x_. During the NO_x_ pauses, the normalized sensor response of S1 remained constant during the first three NO_x_ pauses, but later, when S1 was almost fully NO_x_ loaded, *A* decreased again, indicating that some of the formerly stored NO_x_ was released during NO_x_ pauses. Since (practically) no NO_x_ was found in the outlet gas (FTIR spectroscopy data), one has to assume that a “redistribution” of the stored NO_x_ in the catalyst volume occurs. In other words, the released NO_x_ from the material on S1 or the catalyst material around it (indicated by the normalized sensor response decrease during a NO_x_ pause starting at *t* > 1.1 h), must have been restored on storage sites downstream of this position that have not been filled with NO_x_ up to this point in time. Due to the high catalytic activity of the coating, one has to assume that the composition of the released NO_x_ with respect to the NO_2_/NO ratio is in the equilibrium. At 320 °C, the equilibrium ratio of NO_2_/NO is about 70/30 [32]. After the seventh NO_x_ pulse, the normalized sensor response of S2 also starts to change stepwise from the unloaded to the NO_x_ saturated state. But the normalized sensor response of S2 (being downstream of S1) increases further in the NO_x_ pause—in contrast to that of S1. This might also be an indicator for the rearrangement of the NO_x_ stored in the catalyst in NO_x_ pauses: NO_x_ released in the front part can be restored in the rear part of the catalyst with unoccupied storage sites being available. The normalized sensor response of S3 appears to just start changing its value at the end of this experiment. This agrees with the observation that no NO_x_ slip occurs during these 14 NO_x_ pulses.

The total amount of stored NO during this test is calculated as described by Equation (4) and shown in Figure 7(c). The stored NO mass increases stepwise since NO was added to the feed gas in 14 steps and no NO_x_ breakthrough was detected by the FTIR spectrometer.

The resonance frequency, which is related to the amount of stored NO_x_ according to Figure 3(c), is shown in Figure 7(d), again with an inverted ordinate. At first glance, it shows the expected behavior—a continuous increase during each NO_x_ loading phase and a constant value during NO_x_ pauses. By comparing the curve of the total stored NO mass in Figure 7(c) with the resonance frequency data, it seems as if the non-continuous stepwise NO_x_ loading of the catalyst can also be monitored by the microwave-based technique. In contrast to the impedimetric NO_x_ loading sensors installed along the catalyst axis, it cannot measure the NO_x_ loading locally. Therefore one would not have expected any kind of NO_x_ rearrangement in the catalyst volume occurring during the NO_x_ pauses to show up in the microwave signal. Since no NO_x_ was detected by the FTIR spectrometer in the outlet gas, it is excluded that NO_x_ left the catalyst. Hence, the slight resonance frequency increases occurring during NO_x_ pauses between the NO_x_ loading periods must be attributed to either a local rearrangement reaction or to the involvement of different storage reactions (e.g., nitrite-nitrate conversion) [9].

Altogether, the NO_x_ loading sensors are able to give spatially resolved information on the NO_x_ loading state, whereas the perturbation method mainly yields the average state of the catalyst volume. However, with respect to an exact NO_x_ loading detection, the fact that the resonance frequency changes in the absence of NO_x_ in the exhaust gas although the total mass of NO_x_ stored in the LNT material was constant has to be seen very critically.

**Figure 8.**
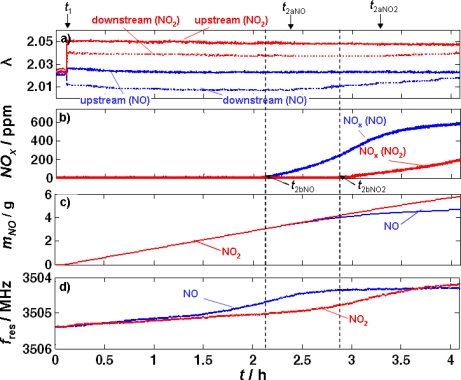
Comparison of NO loading test and NO_2_ loading test, both equal in duration of loading, NO_x_ concentrations, and temperature. The figure shows **(a)** the λ values from UEGO sensors, **(b)** the NO_x_-FTIR spectroscopy data, **(c)** the calculated stored amount of NO and **(d)** the resonance frequencies.

In order to distinguish between the influence of NO and NO_2_ in the feed gas, two separate experiments were carried out. In both gases, the LNT was kept at 320 °C and it was NO_x_ loaded for 4 h. Either 750 ppm of NO or NO_2_ were added to the lean base gas. The NO or NO_2_ dosing started at *t*_1_ in both cases. The measurement results are compared in Figure 8.

In Figure 8(a), λ upstream and downstream of the catalyst obtained from the UEGO sensors is plotted. In the case of NO-dosing, λ upstream of the LNT jumps from 2.020 to 2.022 [indicated by “upstream (NO)”] as soon as NO is admixed to the base gas at *t*_1_. In contrast, λ rises up to 2.045 when NO_2_ is added instead of NO [“upstream (NO_2_)”. Downstream of the LNT, λ is 2.016 if NO and 2.038 if NO_2_ is admixed [“downstream (NO)” and “downstream (NO_2_)”, respectively]. The differences in λ downstream of the catalyst during NO or NO_2_ exposure can be explained the following: The catalytically active precious metal particles of the LNT catalyze the equilibrium reaction of NO and NO_2_. As described earlier, the NO_2_/NO-ratio is about 70/30 at 320 °C. In the case of the addition of NO to the feed gas, NO needs to be oxidized. Since this reaction consumes O_2_, λ decreases after adding NO. The opposite happens if one adds NO_2_ to the base gas: O_2_ is produced due to the reduction of some NO_2_ to NO and λ downstream of the catalyst increases. In the course of the experiment, the signals of the UEGO sensors downstream of the catalyst started to increase noticeably. In the case of NO exposure this occurs approximately at *t*_2aNO_ ≈ 2.4 h and at *t*_2aNO2_ ≈ 3.3 h if NO_2_ is added to the base gas. Obviously, NO_x_ slip starts to occur earlier if NO instead of NO_2_ is dosed.

This observation is confirmed by the FTIR gas analysis concentration downstream of the catalyst [Figure 8(b)]. Here, in both experiments NO_x_, was detected a little earlier (*t*_2bNO_ and *t*_2bNO2_, respectively) compared to the increase in λ. The NO_2_/NO-ratio measured by the FTIR spectrometer was at about 70/30 in both cases, which agrees with the theoretically expected values [32], proving the high catalytic activity of the catalyst. The increase of the NO_x_ levels when the first NO_x_ slip occurs is higher if only NO is added to the base gas than it is in the case of NO_2_ admixture.

In Figure 8(c), the total mass of stored NO calculated from Equation (4) is displayed. Until the first NO_x_ slip occurs at *t*_2bNO_, the lines corresponding to the dosing of NO and NO_2_ are the same and the slope is constant.

Figure 8(d) compares the resonance frequencies of both experiments. The resonance frequency decreases during NO as well as during NO_2_ exposure. At the early stage of the experiment, the resonance frequency decreases slightly faster in the case of loading with NO than with NO_2_. The slope of the curve when only NO is added to the feed gas increases with time, but approximately at *t*_2bNO_ the curve starts to flatten again and a plateau establishes. This behavior is different if only NO_2_ is admixed to the feed gas. Then, an almost constant but small decrease of the resonance frequency can be observed for about 2 h of NO_2_ exposure, later the slope increases for about 0.5 h and flattens as well. It seems that the plateau corresponding to a constant NO_x_ loading begins to settle at the end of the experiment. The plateau in the case of NO_2_ is at slightly lower values than that of NO, indicating a higher storage level, which is in accordance with the data in Figure 7(c).

The catalyst properties determined from the storage [Figure 8(b)] as well as the time characteristics of λ downstream of the LNT agree well with the literature [10]. It is well known that the NO_x_ storage properties of LNTs are enhanced if exposing the catalyst to NO_2_ instead of NO and that therefore NO_x_ slips occur at an earlier stage in the case of NO [10].

Looking at the outlet NO_x_ concentration [Figure 8(b)] and the corresponding total stored NO_x_ mass in Figure 8(c), it is obvious that the amount of NO_x_ stored in the catalyst is the same for both experiments until NO_x_ slip occurs at *t*_2bNO_. Up to this point, the average loading state of the catalyst in the case of NO dosing is equal to those during admixed NO_2_ in the feed gas. The curves of the resonance frequencies [Figure 8(d)] already differ from each other at this early stage, which suggests that the NO_x_ loading state of the catalyst is not mirrored correctly in the microwave cavity resonance frequency.

Due to the high catalytic activity of the precious metals in the LNT, one would have expected that NO and NO_2_ are almost in an equilibrium and, therefore, the LNT should have behaved the same, independently of whether NO or NO_2_ is the basis for the nitrate formation. However, these effects have already been addressed in the literature, for instance in [9] or [33]. It has been shown that the special distribution of the NO_x_ molecules stored in the catalyst is different when NO or NO_2_ is in the feed [33] and it has been suggested that different NO_x_ storage reactions prevail in NO_2_ [9]. It is not the aim of this paper to study catalyst chemistry, but it is clear that such effects also may affect the conductivity of the involved materials and therefore influence the microwave parameters.

## 5. A Comparison of the Microwave-Based Approach for LNT NO_x_ Loading Detection with other Exhaust Gas Aftertreatment Devices

The comparative analysis of the microwave data, the λ values, and the FTIR spectroscopy data reveals that the microwave cavity resonance frequency does not unambiguously depend on the average NO_x_ loading state of the LNT as calculated from the gas analysis data. The results suggest that the involved storage reactions and the spatial distribution of the stored NO_x_ in the catalyst depend on the type of NO_x_ dosing. This might be mainly due to a better NO_2_ incorporation in the front area of the catalyst, since in the rear part of the catalyst NO and NO_2_ are in equilibrium owing to the high catalytic activity of the precious metals in the catalyst. The microwave-based method appears to be influenced by these local distributions and, therefore, may not be adequate to monitor the instantaneous average NO_x_ loading state of the LNT *in situ*.

Two of the most crucial parameters of any measurement system for the loading states of catalysts in exhaust gas aftertreatment systems are the sensitivity and the cross-sensitivity. In the case of the microwave-based system, a normalized sensitivity may be defined by:

(6) $$S_{\text{load}}=-\frac{\Delta f_{\text{res}}}{f_{\text{res},\text{unloaded}}}\cdot \frac{10^{6}}{100}$$

where Δ*f*_res_ is the fractional resonance frequency change between the fully loaded state and the unloaded state, the latter being characterized by the frequency *f*_res, unloaded_. The unit of *S*_load_ is ppm/%, *i.e.*, 1 ppm fractional frequency shift per 1% relative load of NO_x_, NH_3_, O_2_, or soot stored in the catalyst or filter, for LNTs, SCR-catalysts, TWCs, or DPFs, respectively.

As can be inferred from Figure 7, the frequency difference between the unloaded and the fully loaded state is about Δ*f*_res_ ≈ −4 MHz and the resonance frequency at 0% NO_x_ load is *f*_res,unloaded_ ≈ 3,496 MHz. This yields *S*_load_ ≈ 11.4 ppm/%. In other words, for the investigated temperature and an O_2_ and NO_x_ concentration in the feed gas as given above, each percent NO_x_ loading (compared to the fully loaded state) reduces the resonance frequency by 11.4 ppm. For ammonia SCR catalysts, data are available from [23]. Here, some frequencies exist, at which water has only a negligible influence, e.g., at about 1,623 MHz. Under the conditions of reference [23] (Table 1; *T* = 300 °C; saturation at 500 ppm loading assumed as 100% ammonia load), a sensitivity of *S*_load_ ≈ 46.58 ppm/% is observed. By far larger sensitivities occur for the TWC and DPF cases. If one considers a fully soot loaded DPF of 9.2 g/L, one obtains a resonance frequency shift of about 60 MHz at a resonance frequency of ca 1,370 MHz [21]. This leads to a sensitivity of *S*_load_ ≈ 438 ppm/%. An effect of similar magnitude is found in TWCs. In this case, the resonance frequency changes very selectively (no cross effects towards H_2_O, CO, CO_2_ and only a very slight one towards the gas flow velocity) between 1,235 MHz in the rich oxygen-depleted case and 1,270 MHz in the fully oxidized lean case, corresponding to a sensitivity of *S*_load_ ≈ −276 ppm/% [35].

It should be noted at this point that it is very difficult to define 100% full load, since it mostly depends on the gas concentration, as this is also the case for LNT [*cf.* Figure 3(b); there is an additional storage after increasing the inlet NO concentration from 2,000 to 4,000 ppm]. Therefore, the *S*_load_ values are only rough estimates, but the trend that the sensitivity in the case of the LNT is much lower remains. This is attributed to the fact that, despite the conductivity increase of the NO_x_ storage material due to the NO_x_ loading, the material always remains a bad conductor [19,29,36], especially when compared with the conductivity changes in the case of TWC coating material or of soot deposited in DPFs.

In TWCs, ceria-zirconia mixed oxides in the washcoat serve as oxygen storage components. Ce changes its valence state from Ce^4+^ to Ce^3+^ when it gets reduced and back to Ce^4+^ when it gets oxidized. Since the concentration of Ce^3+^ is identical with the free electron concentration, the conductivity of the TWC coating responds strongly to oxygen loading [37]. This effect is so pronounced that even conductometric oxygen gas sensors using ceria zirconia mixtures are under investigation [38]. The difference in conductivity between the oxygen-depleted and the oxygen-loaded states is about three decades. Small side effects stemming from responses towards NO_2_ or CO, as they can typically be expected for n-type semiconductors [39], occur in the small region around the interface between grains and exhaust and barely affect the overall conductivity. Hence, the microwave signal hardly responds to these possible interfering gas components [35].

In DPFs, soot is deposited in the filter channels of the ceramic wall flow filters [40]. Since soot is electrically conducting—the electrical surface conductivity of planar alumina substrates changes by decades during soot deposition [41,42]—the microwave properties are strongly affected. The effects are large, since already a small soot load leads to significant microwave absorption and resonance frequency shifts [21], and disturbing effects are negligible.

In ammonia SCR catalysts, the knowledge of the ammonia loading is crucial, especially at low catalyst operation temperatures. The importance of the ammonia loading on zeolite-based SCR-catalyst has been shown for example in reference [43]. Although the resonance shift is smaller than in the case of DPFs and TWCs and only a little larger than in the case of LNTs, one cannot compare the effects occurring for LNTs and zeolite-based SCR catalysts, as there is a significant difference in the mechanism that leads to conductivity increases in these lean NO_x_ abatement systems. In the zeolites, the conductivity is an ionic one, and owing to the framework structure in combination with the assumed Grotthus type conduction mechanism [44], a very selective conductivity change occurs with ammonia loading [24,45]. Clearly, a strong temperature dependency has to be expected in the case of zeolite-based SCR catalysts, but compared to the LNT, at least there is no cross-sensitivity towards the oxygen concentration [see the differences between lean and rich in Figure 3(c)]. In addition, in the TWC and DPF systems, the reproducibility is high. For instance, in the study of DPFs in Reference [21], seven different samples were investigated and all samples behaved in a very similar manner. In the present study, only one LNT device was used. Small changes in the setup and maybe also in the sample temperature lead to a shift in the resonance frequencies that exceeds the measuring effect. This can be seen for instance in the small difference of the resonance frequencies in the unloaded state between Figures 7 and 8. Furthermore, from impedimetric measurements of the LNT materials, a strong temperature dependence is known [34]. Hence, one can expect that the microwave signals in LNTs are by far more affected by temperature than they are for instance in soot-loaded DPFs.

## 6. Conclusion

Two powerful *in-situ* measurement techniques—one wirebound and one microwave-based—were applied to a NO_x_ storage and reduction catalyst (LNT) to obtain further knowledge about the reactions occurring in the catalyst volume. As a result of previous research on other catalyst systems (three-way catalyst, SCR systems and diesel particulate filters) the signals of the microwave-based approach were expected to mainly reflect the average loading state of the whole catalyst volume, whereas the wirebound sensors were assumed to locally resolve it.

It was shown that both systems are able to detect the different catalyst conditions “lean without NO_x_” and “lean with NO_x_” as a result of differences in the electrical properties of the storage material.

In addition to the microwave-based setup, three impedimetric sensors—coated with the catalyst material—were inserted in the catalyst device equidistantly. With this combined setup, the progression of the NO_x_ loading front along the catalyst during NO_x_ in the feed could be monitored simultaneously with the microwave-based as well as with the wirebound technique. The measurement results with the impedimetric sensors demonstrated that the NO_x_ loading starts in the front part of the catalyst volume and propagates downstream. The stepwise loading of the catalyst by switching between lean gas with and without NO_x_ could be seen in the microwave data as well as in the sensor response. The sensor signals showed additionally that there is a reordering of the formerly stored NO_x_ in the absence of NO in the feed gas. This release and restoring process yields a broadening of the storage profile in the catalyst. The resonance frequency appears to be also influenced by this reordering, which might be caused by the differences in the distribution of the stored NO_x_ in the catalyst. In addition, the influence of the kind of NO_x_ dosing was investigated: the curve of the gas analysis data showed that NO_2_ can be stored more easily and to a higher extent in the catalyst volume compared to NO. The resonance frequency curve also reflected this up to a certain extent of loading. But already prior to the first NO_x_ slip, the resonance frequency shift decreased nonlinearly and deviated from the loading state of the catalyst calculated by balancing the mass of stored NO. This effect became very pronounced when the influence on the microwave signal was studied for separate admixtures of NO or NO_2_ to the lean feed gas. The differences in the resonance shifts cannot be explained by a different NO_x_ loading level of the LNT.

As a conclusion, our experiments indicate that in general the NO_x_ loading state of an LNT can be detected by the microwave-based technique. However, the sensitivity of the method is small compared to that previously observed for three-way catalysts, SCR catalysts and diesel particulate filters. For this reason, interfering effects gain in importance, which may limit the practical applicability of the microwave-based NO_x_ loading detection in lean NO_x_ traps.
